# Alleviation of drought stress through foliar application of thiamine in two varieties of pea (*Pisum sativum* L.)

**DOI:** 10.1080/15592324.2023.2186045

**Published:** 2023-03-08

**Authors:** Abida Kausar, Noreen Zahra, Hina Zahra, Muhammad Bilal Hafeez, Sara Zafer, Abida Shahzadi, Ali Raza, Ivica Djalovic, PV Vara Prasad

**Affiliations:** a Department of Botany, Government College Women University, Faisalabad, Pakistan; b Department of Botany, University of Agriculture, Faisalabad, Pakistan; c Department of Agronomy, University of Agriculture, Faisalabad, Pakistan; d Department of Botany, GC University, Faisalabad, Pakistan; e Department of Economics, Government College University, Faisalabad, Pakistan; f College of Agriculture, Fujian Agriculture and Forestry University (FAFU), Fuzhou, China; g Institute of Field and Vegetable Crops, National Institute of the Republic of Serbia, Novi Sad, Serbia; h Department of Agronomy, Kansas State University, Manhattan, KS, USA

**Keywords:** Abiotic stress, antioxidants, biochemical attributes, legume crop, pea breeding, Vitamin B1

## Abstract

Drought stress poorly impacts many morphological and physio-biochemical processes in plants. Pea (*Pisum sativum* L.) plants are highly nutritious crops destined for human consumption; however, their productivity is threatened under drought stress. Thiamine (vitamin B1) is well-known essential micronutrient, acting as a cofactor in key metabolic processes. Therefore, this study was designed to examine the protective effect of foliar application of thiamine (0, 250, and 500 ppm) on two varieties of pea plants under drought stress. Here, we conducted the pot experiment at the Government College Women University, Faisalabad, to investigate the physio-biochemical and morphological traits of two pea varieties (sarsabz and metior) grown under drought stress and thiamine treatment. Drought stress was applied to plants after germination period of 1 month. Results showed that root fresh and dry weight, shoot fresh and dry weight, number of pods, leaf area, total soluble sugars, total phenolics, total protein contents, catalase, peroxidase, and mineral ions were reduced against drought stress. However, the application of thiamine (both 250 and 500 ppm) overcome the stress and also enhances these parameters, and significantly increases the antioxidant activities (catalase and peroxidase). Moreover, the performance of sarsabz was better under control and drought stress conditions than metior variety. In conclusion, the exogenous application of thiamine enabled the plants to withstand drought stress conditions by regulating several physiological and biochemical mechanisms. In agriculture, it is a great latent to alleviate the antagonistic impact of drought stress on crops through the foliar application of thiamine.

## Graphical abstract

**Figure:**
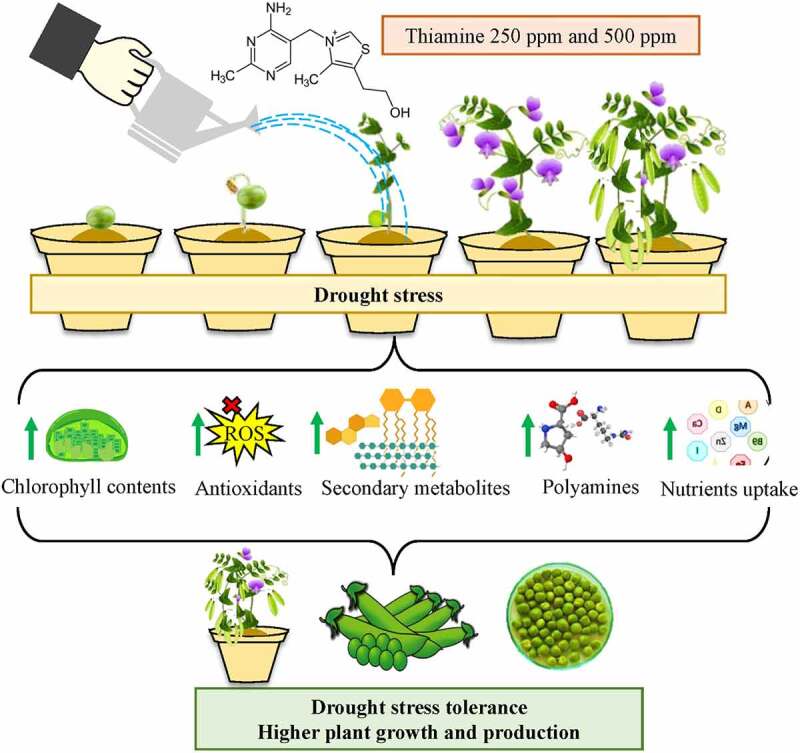

## 1. Introduction

Water, an essential element of cell protoplasm, plays a crucial role in sustaining all life on earth; however, periodic climate shifting limits water accessibility, causing serious problems, e.g., the present water-threatened regimes in various areas of the biomass.^1–4^ Water limitation is one of the most serious problems in many countries, particularly in Pakistan^5^. It adversely deteriorates the plant growth and agriculture yield production of many traditional crops by affecting physiological responses, including osmotic adjustment, photosynthesis, transpiration, and carbon metabolism.^6–8^ In addition, physio-biochemical parameters such as enzyme activities, electrolyte leakage, hydrogen peroxide, lipid peroxidation, antioxidant machinery, proline content, and relative water content were adversely affected under drought stress.^9–12^ Water status understanding plays an important role in securing the food production for rapidly overgrowing population by supporting sustainable agriculture production.^12,13^ Gene regulatory network revealed that 69 hub-genes, including independent and integrated ABA-dependent pathways controlling the sense of drought, senescence, stomatal closure, antibiotics pathways, thiamine metabolism, purine metabolism, uptake regulation and root growth.^14^ Grain legumes are highly recognized due to their seeds with high nutritional value. To response the rising concerns about protein autonomy and food security, grain legume cultivation must be increased in up-coming years.^15^ Pea (*Pisum sativum* L.) is among the most cultivated pulse corps across the world. Its economic value is particularly obtained from its nutritious seeds having low fat, and high dietary fibers, vitamins, essential minerals, proteins, carbohydrates, iron, zinc, and slow-digestible starch.^16–18^ Agriculture productivity is facing environmental challenges, so like many other crops, pea is predominantly prone to abiotic stresses, especially more and severe episode of drought that can lead to huge yield losses^19^ that primarily arises from concentrated pollination, seed set, abortion of flower and pods, and shortened life cycle under drought.^20,21^ So, increasing the productivity of pea plants under uncertain conditions of drought stress is an important target.

A diverse range of plant growth-promoting agents accelerates the plant growth and developmental process, and helps plants to thrive under stressful conditions.^22–26^ Recent studies exposed the dual role of vitamins besides their nutritional importance. Vitamins, especially ascorbic acid, and thiamine play influential roles in combating environmental stresses.^27^ Plant-derived vitamins regulate plant metabolism, change the redox chemistry and act as enzymatic cofactors,^28^ therefore enhancing the crop yield and resistance against biotic and abiotic stresses. Thiamine, a growth stimulant that acts as a cofactor in many dynamic plant mechanisms.^29^ It also acts as an antioxidant to alleviate reactive oxygen species (ROS)^30^ and is elaborated in energy-producing mechanisms such as Kreb’s and Calvin cycles.^31^ Respiration and enzymatic activity are also linked with thiamine levels.^32^ Moreover, growth, photosynthetic pigments, total sugars, proteins, antioxidant machinery and yield attributes are remarkably improved with the exogenous application of thiamine.^33,34^ Exogenous application of thiamine enhanced stress tolerance by activating stress-responsive genes and calcium signal transduction.^35^ Furthermore, it also increased the amino acid content, total free amino acids, proline, soluble sugars, photosynthesis, polypeptides, polyehnol oxidase isozymes, peroxidase, growth, and yield attributes under drought stress.^36^ Moreover, overexpression of *thiamine thiazole synthase* (needed for the thiamine biosynthesis) gene proved to be more sensitive to abscisic acid than wild plants with respect of both stomatal closure and activation of guard cell slow type anion channels that resulted in lower transpiration loss of water and enhanced drought tolerance.^37^ Only a few focused studies are present that elucidate the inevitable role of thiamine under drought stress. The present study elaborates on the plausible role of thiamine foliar application in alleviating drought stress in two varieties of pea.

## 2. Materials and methods

### 2.1. Plant material and stress treatment

This research was performed at the research area of Government College Women University, Faisalabad (31.4504° N, 73.1350° E), Pakistan in 2019, to examine the positive role of the exogenous application of thiamine on two pea verities (metior and sarsabz) that were obtained from Ayyub Agricultural Research Institute (AARI), Faisalabad. Ninety-six pots of 8 kg soil capacity were filled with soil, and pots were arranged with completely randomized design (CRD). Four replicates per treatment were used during the experiment. Field capacity of the soil was maintained 50% and 100% after 20 days of germination throughout the experiment. Soil of pot was sandy loam having following properties: pH 7.8, electrical conductivity 2.16 dS m^−1^, organic matter 0.76%, total nitrogen 0.029%, available phosphorus 4.23 mg kg^−1^, and extractable potassium 314 mg kg^−1^. Three levels of thiamine solution were applied as 0, 250, and 500 ppm applied through foliar application after developing drought stress. Estefan^38^ procedure was followed for analysis of site-soil physiochemical properties and nutrient status. Plants leaves were harvested after 3rd stage of BBCH growth scale (a scale which is used to identify the plant’s phenological and developmental stages) to investigate biochemical and growth-related attributes. Half set of experiments was harvested at the yield stage.

### 2.2. Measurement of growth attributes

Shoot length (cm), root length (cm), shoot fresh and dry weight (g), and root fresh and dry weight (g) were measured for four replicates per treatments. In contrast, numbers of pods per plant, pods length, and number of seeds per pod were measured at the maturity stage.

### 2.3. Measurement of biochemical attributes

#### 2.3.1. Total chlorophyll and carotenoids contents

Plant total chlorophyll a, chlorophyll b, and carotenoid contents were measured according to Arnon.^39^ Briefly, a 0.5 g of plant leaf samples were taken and ground in 10 mL of 80%. Then, the samples were centrifuged at 12,000 rpm for 12 min. Readings were taken at 663, 645, and 480 nm on a spectrophotometer. Davies and Goodwin^40^ protocol was used to calculate the total carotenoids contents.

#### 2.3.2. Total phenolic contents

Julkunen-Tiitto^41^ method was used to calculate the total phenolics content in plant leaves. Briefly, a 0.5 g plant leaf sample were ground in 10 mL of 80% solution of methanol. Samples were then centrifuged at 10,000 rpm for 10 min, then 0.25 mL of sample was mixed with 1.25 mL of Folin-Phenol Ciocalteus reagent. In last step, 1.25 mL of sodium carbonate was added, and vortex vigorously for 5–10 s. Then, absorbance of these samples was recorded at 750 nm.

#### 2.3.3. Proline

Total proline content was measured according to Bates.^42^ In brief, a 0.5 g plant leaf sample was taken and mixed with 3% sulfosalicylic acid. Then, running samples were centrifuged at 10,000 rpm for 10 min. For 2 mL of extract, 2 mL of acid ninhydrin were added. The resultant solution was mixed with 2 mL of glacial acetic acid, and incubated at 100°C for 1 h and then cooled in an ice bath. Then, 4 mL of toluene was added to each sample and the sample were vortexed for 1 min. These samples were then subjected to a spectrophotometer to measure the absorbance at 520 nm.

#### 2.3.4. Total soluble sugars

Yemm and Willis^43^ method was used to govern the total soluble sugar contents. Briefly, a 0.1 g plant fresh leaves were taken and then ground in 10 mL of 80% solution of ethanol. The resultant solution was then subjected to centrifugation at 10,000 rpm. Supernatant extract was filtered for estimation of total sugar content. A 0.1 mL of plant extract was taken in separate tubes and 3 mL of anthrone reagent was mixed in each test tube. Then, samples were heated in a water bath for 10 min after ice cooling, and optical density was recorded at 625 nm.

#### 2.3.5. Total protein content

Bradford^44^ method was utilized to evaluate the total soluble protein contents in plant leaves. Briefly, a 0.5 g of fresh plant leaves were ground in 10 mL of buffer solution. The resultant extract was then centrifuged at 10,000 rpm for 10 min. Then, 2 mL of Bradford reagent was poured into each test tubes. Then, 100 µL extract was added in these test tubes. Optical density was observed at 595 nm by using a spectrophotometer.

#### 2.3.6. Antioxidant enzyme

A 0.5 g of fresh plant leaves were taken, then grind in 80 mL of 50 mM phosphate buffer having pH 7.8. After that the sample was centrifuged at 15,000 rpm for 10 min. Supernatants were used for the determination of catalase and peroxidase. Chance and Maehly^45^ method was to estimate plant catalase activity. It was determined by calculating the reduction in absorbance after every 20 s for 120 s at 240 nm. For 2.8 mL of phosphate buffer, 0.1 mL of H_2_O_2_ was added. Further, 0.1 mL of enzyme extract was then poured and slowly shaken well to mix it thoroughly and then observed in a spectrophotometer.

#### 2.3.7. Peroxides

Peroxidase activity was recorded of leaf material by following the method of Chance and Maehly.^45^ For 2.7 mL of phosphate buffer, 0.1 mL of guaiacol and 0.1 mL of enzyme extract was added. Optical density was measured at 470 nm after each 20 s for 120 s collectively.

#### 2.3.8. Ions contents

Wolf^46^ method was used to determine the ions concentration of leaf material. A 0.15 g of dried samples were mixed with 3 mL of sulfuric acid and left overnight. Samples were then heated in a heat bath at 100°C for 30 min. Test tubes were then removed from the heating chamber. This procedure was repeated until the mixture became colorless. Volume of each test tube were then made up to 50 mL with distilled water. Readings of ions analysis sodium (Na^+^), calcium (Ca^2+^), and potassium (K^+^) were noted on flame photometer.

### 2.4. Statistical analysis

The data were statistically analyzed by using statistics 8.1 software (Analytical Software, Tallahassee, FL), and 3-way ANOVA was used to evaluate the significant differences between the treatments. Four replications were used in CRD factorial design with an alpha value 0.05. LSD test was used to compare the treatment means. R-studio software (Ross Ihaka and Robert Gentleman, USA) was used for correlation and PCA analysis. In PCA, spectral decomposition was examined for the covariances/correlations between different variables.

## 3. Results

### 3.1. Growth and yield attributes

#### 3.1.1. Shoot length

Thiamine treatment at 500 ppm significantly increased shoot length irrespective of varietal differences under normal irrigation as well as drought stress. There was a significant interaction between variety and stress factor. In sarsabaz variety, 57.33% and 61.81% increase in shoot length was noted with thiamine spray (500) as compared to control, irrespective of stress treatments control under normal and drought stress conditions, respectively. In metior variety, thiamine spray (500) exhibited 59.80% and 60% increase in shoot length as compared to control irrespective of stress treatments control under normal and drought stress conditions, respectively. Moreover, higher shoot length was seen at 500 ppm of thiamine compared to 250 ppm. Drought stress significantly reduced the shoot length irrespective of varietal differences (Table 1).

**Table 1.** Effect of thiamine and drought stress on growth attributes of two pea varieties. Shoot length, root length, shoot fresh weight, shoot dry weight, root fresh weight, root dry weight, and number of leaves.

Varieties	Thiamine	No. of leaves		Root dry weight (g)		Root fresh weight (g)		Shoot dry weight (g)		Shoot fresh weight (g)		Root length (cm)		Shoot length (cm)	
		Control	Drought	Control	Drought	Control	Drought	Control	Drought	Control	Drought	Control	Drought	Control	Drought
Sarsabz	0 ppm	22.0d	18.6de	0.023f	0.020fg	0.28f	0.21 g	0.28d	0.27d	1.20 g	1.00gh	4.17f	2.73 g	11.67f	8.40gh
	250 ppm	35.33b	30.6c	0.045cd	0.032e	0.44cd	0.38e	0.39bc	0.28d	2.42d	2.02e	6.50bc	5.20de	18.67c	13.67de
	500 ppm	48.0a	38.0b	0.071a	0.054b	0.66a	0.54b	0.53a	0.38c	3.94a	3.15b	9.23a	7.00b	27.33a	22.00b
Metior	0 ppm	17.6ef	14.3f	0.016gh	0.014h	0.18g	0.17g	0.20e	0.13f	0.85hi	0.68i	3.07g	2.27g	7.50hi	6.00i
	250 ppm	27.6c	20.0de	0.038e	0.025f	0.32f	0.29f	0.20e	0.19e	1.77ef	1.60f	5.07e	4.07f	12.00ef	9.50g
	500 ppm	35.6b	29.6c	0.048c	0.043d	0.49c	0.39de	0.42b	0.28d	2.84c	2.46d	7.00b	5.97 cd	18.67c	15.00d

Note: The alphabets indicate the significant (*P* < 0.05) difference among the treatments.

#### 3.1.2. Root length

Thiamine treatment (500 ppm) had higher root lengths in plants under normal irrigation as well as drought stress. Interaction between stress and varieties was significant. In sarsabaz variety, thiamine foliar application at 500 ppm showed 54.92% upsurge in root length than control irrespective of stress treatments control conditions. In metior variety, higher dose of thiamine showed 56.28% increase in root length in plants than untreated plants irrespective of stress treatments control under normal conditions, while under drought stress conditions, 62.08% increase in root length was noted as compared to control irrespective of stress treatments control plants. Moreover, root length improved with an increasing dose of thiamine (Table 1).

#### 3.1.3. Shoot fresh weight

Thiamine treatment at 500 ppm significantly improved the shoot fresh weights under normal irrigation and drought stress. Treatment and stress factors had more significant interaction between them. In sarsabaz variety, 500 ppm thiamine increased 69.54% and 70.96% shoot fresh weight than control irrespective of stress treatments control under normal and drought conditions, respectively. In metior variety, 500 ppm thiamine increased 64.28% and 73.33% shoot fresh weight than control irrespective of stress treatments control under normal and drought conditions, respectively. Moreover, higher shoot fresh weight was seen at 500 ppm of thiamine as compared with 250 ppm (Table 1).

#### 3.1.4. Shoot dry weight

Thiamine treatment at 500 ppm in T3 had significantly increased shoot dry weights under normal irrigation and drought stress. There was more significant interaction between treatment and stress. However, stress, varieties and treatment had non-significant interactions between them. In sarsabz variety, 500 ppm thiamine increased 48.07% and 52.63% shoot dry weight as compared to control irrespective of stress treatments control under normal and drought conditions, respectively. In metior variety, 500 ppm thiamine increased 50% and 44.44% shoot dry weight than control irrespective of stress treatments control under normal and drought conditions, respectively. Moreover, higher shoot dry weight was seen at 500 ppm of thiamine as compared with 250 ppm (Table 1).

#### 3.1.5. Root fresh weight

Thiamine treatment at 500 ppm had higher root fresh weights under normal irrigation as well as drought stress. There was a highly significant interaction between treatment and stress. Pea varieties and stress non-significantly affected root fresh weight. There was a noteworthy interaction among treatment and stress. Higher root fresh weight was seen in sarsabz than metior variety. Moreover, higher root fresh weight was seen at 500 ppm of thiamine as compared with 250 ppm. Drought stress significantly declinethe root fresh weight irrespective of varietal differences (Table 1).

#### 3.1.6. Root dry weight

Thiamine treatment at 500 ppm had higher root dry weights under normal irrigation as well as drought stress. There was a highly significant interaction between treatment and stress. Pea varieties and stress non-significantly affected root dry weight. There was a significant interface among treatment and stress. Higher root dry weight was seen in sarsabz than metior variety. Moreover, higher root dry weight was seen at 500 ppm of thiamine as compared with 250 ppm. Drought stress significantly decreased the root dry weight irrespective of varietal differences (Table 1).

#### 3.1.7. Number of leaves per plant

Thiamine treatment at 500 ppm had a higher number of leaves under normal irrigation and drought stress. There was a highly significant interaction between treatment and stress. Pea varieties and stress non-significantly affected number of leaves. Maximum number of leaves was seen in sarsabz as than metior variety with the application of thiamine at 500 ppm as compared with 250 ppm. Drought stress significantly declined the number of leaves per plant irrespective of varietal differences (Table 1).

#### 3.1.8. Pod length

Thiamine treatment at 500 ppm significantly increased pod length under normal irrigation as well as drought stress. Treatment, stress, and varieties had non-significant interactions between them. In sarsabz variety, 53.99% and 52.79% increase in pod length was noticed with the application of 500 ppm thiamine as compared to control irrespective of stress treatments control under normal and stressful conditions, respectively. In metior variety, 52.66% and 68.38% increase in pod length was detected with the application of 500 ppm thiamine than control irrespective of stress treatments control under normal and stressful conditions, respectively. Maximum pod length was seen in sarsabz as than metior variety with the application of thiamine at 500 ppm as compared with 250 ppm. Drought stress significantly reduced the pod length irrespective of varietal differences (Table 2).

**Table 2.** Effect of thiamine and drought stress on yield-related attributes of two pea varieties. Pod length, number of pods per plant, seed fresh weight, and seed dry weight.

Varieties	Thiamine	Pod length (cm)		No. of seeds pod^−1^		Seed dry weight (g)		Seed fresh weight (g)	
		Control		Drought		Control		Drought	
Sarsabz	0 ppm	4.03de	3.13f	2.17gh	1.70h	0.52d	0.40e	1.30f	1.10f
	250 ppm	6.17b	5.13c	3.33cd	2.67ef	0.81b	0.62c	2.33c	1.83de
	500 ppm	8.77a	6.63b	5.33a	4.27b	1.17a	0.84b	4.18a	3.09b
Metior	0 ppm	2.60f	1.67g	1.87h	1.22i	0.40e	0.28f	0.97f	1.10f
	250 ppm	4.50d	3.90e	2.40fg	2.11gh	0.62c	0.45de	1.77e	1.20f
	500 ppm	6.47b	5.25c	3.62c	3.13de	0.86b	0.61c	3.10b	2.21 cd

Note: The alphabets indicate the significant (*P* < 0.05) difference among the treatments.

#### 3.1.9. Number of pods per plant

Thiamine treatment at 500 ppm significantly increased number of pods per plant normal irrigation as well as drought stress. Treatment, stress, and varieties had highly significant interactions between them. In sarsabz variety, 37.5% and 43.4% increase in number of pods per plant was noticed with the application of 500 ppm thiamine than control irrespective of stress treatments control under normal and stressful conditions, respectively. In metior variety, 41.66% and 63.05% increase in this attribute was observed with the application of 500 ppm thiamine as compared to control irrespective of stress treatments control under normal and stressful conditions, respectively. Maximum number of pods per plant was seen in sarsabz as than metior variety with the application of thiamine at 500 ppm as compared with 250 ppm. Drought stress significantly reduced the number of pods per plant irrespective of varietal differences (Table 2).

#### 3.1.10. Seed fresh weight

Thiamine treatment at 500 ppm significantly increased seed fresh weight under normal irrigation as well as drought stress. Treatment, stress, and varieties had non-significant interactions between them. In sarsabz variety, 68.82% and 64.28% increase in seed fresh weight was noticed with the application of 500 ppm thiamine as compared to control irrespective of stress treatments control under normal and stressful conditions, respectively. In metior variety, 69.03% and 50.22% increase in seed fresh weight was recorded with the application of 500 ppm thiamine as compared to control irrespective of stress treatments control under normal and stressful conditions, respectively. Maximum seed fresh weight was seen in sarsabz as than metior variety with the application of thiamine at 500 ppm as compared with 250 ppm. Drought stress significantly reduced the seed fresh weight irrespective of varietal differences (Table 2).

#### 3.1.11. Seed dry weight

Thiamine treatment at 500 ppm had higher seed dry weight under normal irrigationand drought stress. There was highly significant interaction between treatment and stress. Pea varieties and stress significantly affected this attribute. Maximum seed dry weight was seen in sarsabz as than metior variety with the application of thiamine at 500 ppm as compared with 250 ppm. Drought stress significantly reduced the seed dry weight per plant irrespective of varietal differences (Table 2).

### 3.2. Biochemical attributes

#### 3.2.1. Chlorophyll a content

**Figure 1.**
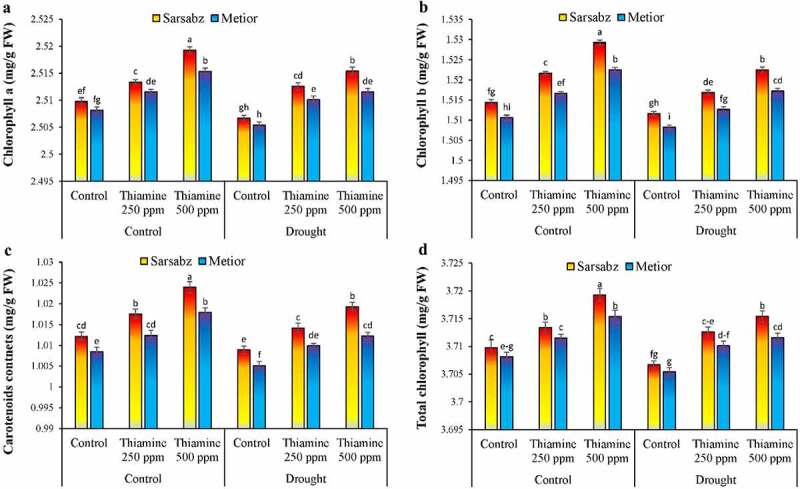
Effect of thiamine and drought stress on photosynthetic pigments of two pea varieties: (a) chlorophyll a, (b) chlorophyll b, (c) carotenoids content, (d) total chlorophyll contents. The alphabets indicate the significant (*P* < 0.05) difference among the treatments.

Thiamin-treated plants had higher chlorophyll under normal irrigation as well as drought stress. A highly significant interaction was noticed among treatment and stress. Pea varieties and stress non-significantly affected this attribute. Maximum increase was seen in sarsabz as than metior variety with the application of thiamine at 500 ppm as compared with 250 ppm. Drought stress significantly reduced this attribute irrespective of varietal differences (Figure 1A).

#### 3.2.2. Chlorophyll b content

Thiamine treatment at 500 ppm had higher chlorophyll b under normal irrigation as well as drought stress. A non-significant interaction was observed between treatment and stress. Pea varieties and stress non-significantly affected this attribute. Maximum increase was seen in sarsabz as than metior variety with the application of thiamine at 500 ppm as compared with 250 ppm. Drought stress significantly reduced this attribute irrespective of varietal differences (Figure 1B).

#### 3.2.3. Carotenoids content

Foliar application of thiamine showed higher carotenoid contents under normal irrigation as well as drought stress. A non-significant interaction was recorded among treatment and stress. Pea varieties and stress non-significantly affected this attribute. Maximum increase was seen in sarsabz as than metior variety with the application of thiamine at 500 ppm as compared with 250 ppm. Drought stress significantly reduced this attribute irrespective of varietal differences (Figure 1C).

#### 3.2.4. Total chlorophyll content

Foliar application of thiamine showed higher total chlorophyll contents under normal irrigation as well as drought stress. There was non-significant interaction between treatment and stress. Pea varieties and stress non-significantly affected this attribute. Maximum increase was seen in sarsabz as than metior variety with the application of thiamine at 500 ppm as compared with 250 ppm. Drought stress significantly reduced this attribute irrespective of varietal differences (Figure 1D).

#### 3.2.5. Proline content

**Figure 2.**
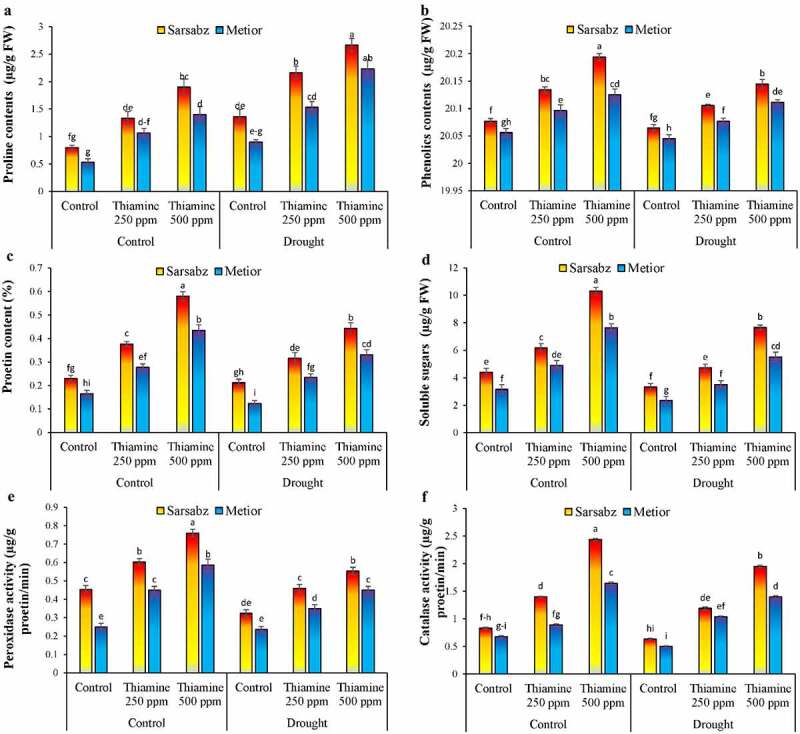
Effect of thiamine and drought stress on secondary metabolites and antioxidant activities of two pea varieties: (a) proline contents, (b) phenolic contents, (c) protein contents, (d) soluble sugars, (e) peroxidase, (f) catalase. The alphabets indicate the significant (*P* < 0.05) difference among the treatments.

Thiamine exogenous application showed higher proline content under normal irrigation as well as drought stress. There was non-significant interaction between treatment and stress. Pea varieties and stress non-significantly affected this attribute. Maximum increase was seen in sarsabz as than metior variety with the application of thiamine at 500 ppm as compared with 250 ppm. Drought stress significantly increased this attribute irrespective of varietal differences (Figure 2A).

#### 3.2.6. Total phenolics

Foliar application of thiamine showed higher total phenolics contents under normal irrigation as well as drought stress. There was a significant interaction between treatment and stress. Pea varieties and stress non-significantly affected this attribute. Maximum increase was seen in sarsabz as than metior variety with the application of thiamine at 500 ppm as compared with 250 ppm. Drought stress significantly reduced this attribute irrespective of varietal differences (Figure 2B).

#### 3.2.7. Total protein content

Thiamine treatment at 500 ppm significantly increased total protein content normal irrigation as well as drought stress. Treatment, stress and varieties had significant interactions between them. In sarsabz variety, 62.06% and 52.27% increase in total protein content was observed with the application of 500 ppm thiamine as compared to control, irrespective of stress treatments control under normal and stressful conditions, respectively. In metior variety, 62.29% and 62.83% increase in this attribute were observed with the application of 500 ppm thiamine as compared to control irrespective of stress treatments control under normal and stressful conditions, respectively. Maximum total protein content was seen in sarsabz as than metior variety with the application of thiamine at 500 ppm as compared with 250 ppm. Drought stress significantly reduced the total protein content irrespective of varietal differences (Figure 2C).

#### 3.2.8. Total soluble sugar content

Thiamine treatment at 500 ppm significantly increased total soluble sugar content normal irrigation as well as drought stress. Treatment, stress, and varieties had non-significant interactions between them. In sarsabz variety, 57.41% and 56.47% increase in total soluble sugar content was observed with the application of 500 ppm thiamine as compared to control irrespective of stress treatments control under normal and stressful conditions, respectively. In metior variety, 58.82% and 57.60% increase in this attribute was observed with the application of 500 ppm thiamine as compared to control irrespective of stress treatments control under normal and stressful conditions, respectively. Maximum total soluble sugar content was seen in sarsabz as than metior variety with the application of thiamine at 500 ppm as compared with 250 ppm. Drought stress significantly reduced the total soluble sugar content irrespective of varietal differences (Figure 2D).

#### 3.2.9. Peroxidase activity

Thiamine exogenous application showed higher peroxidase activity under normal irrigation as well as drought stress. There was non-significant interaction between treatment and stress. Pea varieties and stress non-significantly affected this attribute. Maximum increase was seen in sarsabz as than metior variety with the application of thiamine at 500 ppm as compared with 250 ppm. Drought stress significantly reduced this attribute irrespective of varietal differences (Figure 2E).

#### 3.2.10. Catalase activity

Foliar application of thiamine showed higher catalase activity under normal irrigation as well as drought stress. A non-significant interaction was observed between treatment and stress. Pea varieties and stress non-significantly affected this attribute. Maximum increase was seen in sarsabz as than metior variety with the application of thiamine at 500 ppm as compared with 250 ppm. Drought stress significantly reduced this attribute irrespective of varietal differences (Figure 2F).

### 3.3. Ions contents

#### 3.3.1. Potassium content

**Figure 3.**
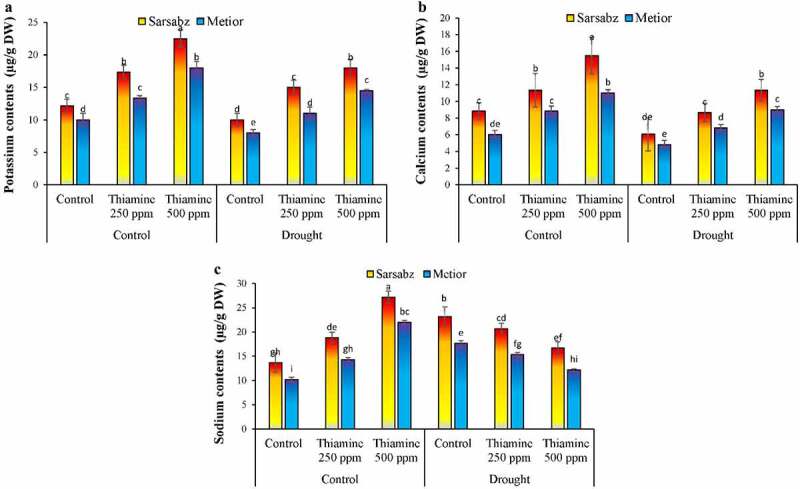
Effect of thiamine and drought stress on ion uptake of shoot of two pea varieties: (a) potassium contents, (b) calcium contents, (c) sodium contents. The alphabets indicate the significant (*P* < 0.05) difference among the treatments.

Thiamine exogenous application showed higher potassium content under normal irrigation as well as drought stress. A non-significant interaction was noticed between treatment and stress. Pea varieties and stress non-significantly affected this attribute. Maximum increase was seen in sarsabz as than metior variety with the application of thiamine at 500 ppm as compared with 250 ppm. Drought stress significantly reduced this attribute irrespective of varietal differences (Figure 3A).

#### 3.3.2. Calcium content

Foliar application showed higher potassium content under normal irrigation as well as drought stress. There was a significant interaction between treatment and stress. Interactions between varieties, stress, and treatments was non-significant. Pea varieties and stress non-significantly affected this attribute. Maximum increase was seen in sarsabz as than metior variety with the application of thiamine at 500 ppm as compared with 250 ppm. Drought stress significantly reduced this attribute irrespective of varietal differences (Figure 3B).

#### 3.3.3. Sodium content

Foliar application showed higher sodium content under normal irrigation as well as drought stress. There was a significant interaction between treatment and stress. Interactions between varieties, stress and treatments was significant. Pea varieties and stress non-significantly affected this attribute. Maximum increase was seen in sarsabz as than metior variety with the application of thiamine at 500 ppm as compared with 250 ppm. Drought stress significantly reduced this attribute irrespective of varietal differences (Figure 3C).

#### 3.3.4. PCA and correlation

**Figure 4.**
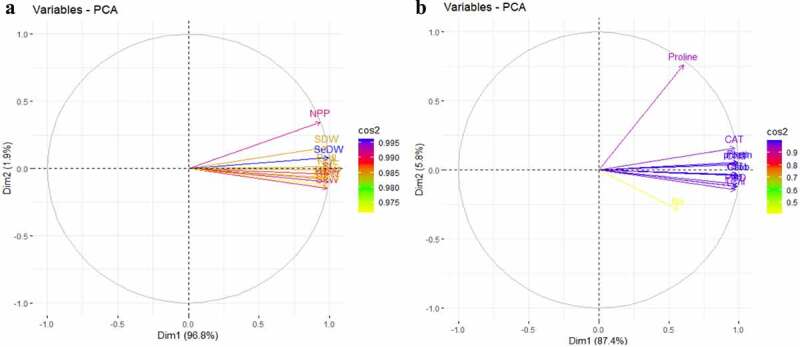
Principal component analysis based on two pea varieties grown under the influence of two thiamine doses and drought stress. First PCA (a) indicates the growth and yield attributes, while second for biochemical attributes (b). The positions of the different variables, includes root length (RL), number of pods per plant (NPP), number of leaves (NOL), pod length (podL), root fresh weight (RFW), root dry weight (RDW), shoot fresh weight (SFW), shoot dry weight (SDW), number of seeds per plant (NSPP), seed fresh weight (SeFW), seed dry weight (SeDW), catalase (CAT), peroxidase (POD), sodium (Na), potassium (K), calcium (Ca), soluble sugars (SS), and phenolics (phenol). As indicated at the bottom left of each circle, (a) gathers 96.8% of the total variance, whereas (b), 87.4% of the total variance.

**Figure 5.**
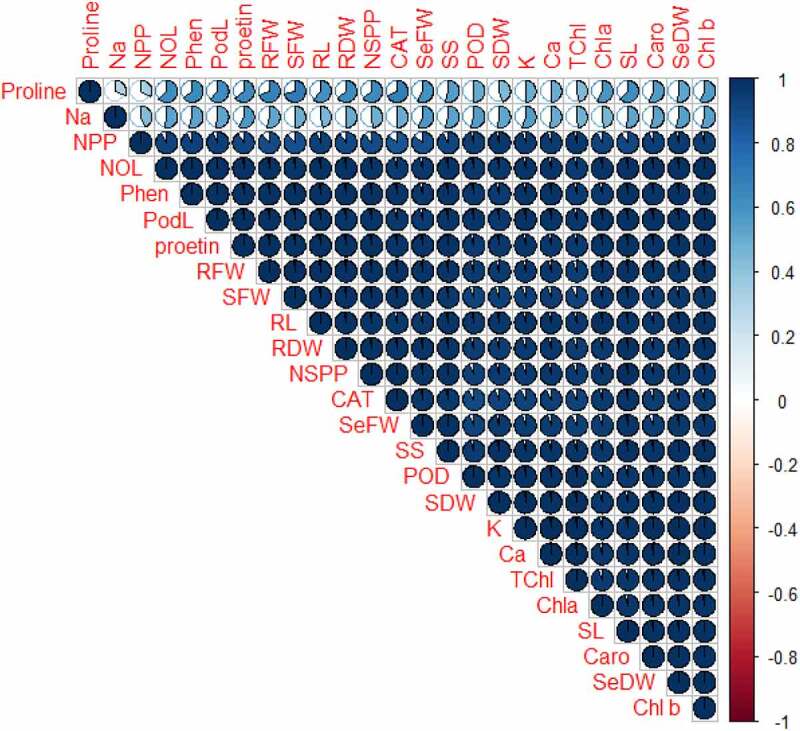
Pearson correlation among different variables in two pea varieties with an application of thiamine under drought stress. The positions of the different variables, includes root length (RL), number of pods per plant (NPP), number of leaves (NOL), pod length (podL), root fresh weight (RFW), root dry weight (RDW), shoot fresh weight (SFW), shoot dry weight (SDW), number of seeds per plant (NSPP), seed fresh weight (SeFW), seed dry weight (SeDW), catalase (CAT), peroxidase (POD), sodium (Na), potassium (K), calcium (Ca), soluble sugars (SS), and phenolics (phenol).

**Figure 6.**
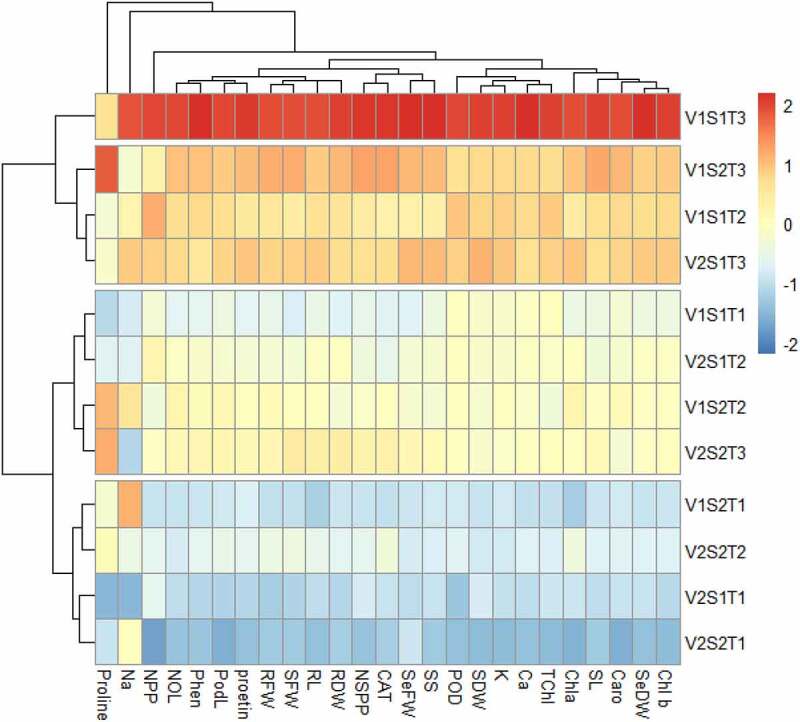
Heatmap matrix among different variables in two wheat varities with application of thiamine under drought stress. V1 and V2 indicates sarsabz and metior varieties, respectively, while S1 and S2 for control and normal conditions. T1, T2, and T3 for control, 250 ppm and 500 ppm thiamine doses. The positions of the different variables, includes root length (RL), number of pods per plant (NPP), number of leaves (NOL), pod length (podL), root fresh weight (RFW), root dry weight (RDW), shoot fresh weight (SFW), shoot dry weight (SDW), number of seeds per plant (NSPP), seed fresh weight (SeFW), seed dry weight (SeDW), catalase (CAT), peroxidase (POD), sodium (Na), potassium (K), calcium (Ca), soluble sugars (SS), and phenolics (phenol).

First, PCA was performed by using the data of all growth and yield-related variables and the second PCA for biochemical attributes, including ion analysis. The first PCA component (PC1) depicted most of the inertia of the data (96.8% of total variance) and showed close relation with all variables (Figure 4a). The PC2 explained 1.9% of the variance of the population, and was mainly driven by the number of pods per plant and shoot dry weight (Figure 4b). The PC1 and PC2 accounted for 87.4% and 5.8% of the total variance and was mainly driven by proline and sodium content. Pearson correlation showed a positive linear correlation among all growth, yield and biochemical attributes, except a negative correlation with sodium and proline content (Figure 5). Heatmap matrix showed that the performance of sarsabz variety with the application of 500 ppm of thiamine was more effective in improving all the parameters (growth, yield, and biochemical) under normal conditions as compared to control irrespective of stress treatments other treatments. Intriguingly, the performance of sarsabz variety was best under control and stressful conditions as compared to control irrespective of stress treatments metior. The metior variety showed negative relation with all parameters under no-spray treatment at drought stress condition (Figure 6).

## 4. Discussion

Drought stress is frequently increasing in the milieu of changing climate and is a foremost constraint for agriculture production. The capacity of plants to sustain their higher productivity under drought stress depends on their capability to endure the drought stress with subsequent recovery.^2,47,48^ This study designed at dynamically assessing the drought response of two pea varieties, namely sarsabz and metior, with the application of thiamine as growth promoting agent. Drought stress led to a remarkable reduction in shoot length, root length, shoot fresh and dry weight, and root fresh and dry weight and number of leaves as shown in Table 1. Our results are in corroborated with the findings of Arafa et al.,^19^ they found that drought stress severely reduced the leaf area plant height and number of leaves per plant as compared with control plants. These outcomes are in agreement with the findings of some investigators in diverse plant species under drought stress, for example, faba bean,^49,50^ sugar beat,^51^ barley^52^ and flax plants.^53^ According to Mohammadi Alagoz et al.,^54^ frequent and intense drought events impact agricultural productionby challenging water status, resulting in lower plant growth and development. However, thiamine applications 250 and 500 ppm effectively stimulate all the growth-related attributes under drought stress. Aminifard et al.^55^ also deduced that thiamine application at the rate of 250, 500 and 700 ppm increased the growth and yield-related attributes in fenugreek and coriander. A similar increase was also confirmed in Table 2. In sarsabz variety, 37.5% and 43.4% increase in number of pods per plant was observed with the application of 500 ppm thiamine as compared to control irrespective of stress treatments control irrespective of stress treatments, respectively. In metior variety, 41.66% and 63.05% increase in number of pods was observed with the application of 500 ppm thiamine as compared to control irrespective of stress treatments control under normal and stressful conditions, respectively. However, drought stress adversely affected the yield attributes of pea irrespective of varietal differences. Arafa et al.^19^ also noted remarkable reduction in number of flowers per plant, pod length and number of pods per plant in drought exposed pea. Similar trend of reduction was seen in the present trial. As reviewed by Khan et al.,^56^ the accretion of plant growth-promoting agents cope with the stress condition; through contributing detoxification of ROS, osmoregulation and pH adjustment, so higher growth and yield performance was ascertained with the foliar spray of thiamine.

Moreover, drought stress poorly exacerbated the photosynthetic system, and deteriorated the accumulation of assessor pigments such as chlorophyll a, chlorophyll b, and carotenoids (Figure 1a-–d). The reduced photosynthetic efficiency was due to the stomatal closure and downregulation of the photosynthetic pathway and Calvin cycle genes.^57^ However, the application of 500 ppm of thiamine efficiently improved the photosynthetic pigments, especially sarsabz as compared with metior varieties, as confirmed by Aminifard et al.,^55^ they found a similar trend with the foliar application of 500 and 700 ppm of thiamine. ABA seems to take part in the genes activation, encoding enzymes that function in thiamine,^58^ which triggered the regulatory pathways to improve physiological responses, which may be the reason of improved photosynthetic activities with thiamine application. The reason for the reduction of chlorophyll pigments is may be associated with the degradation of chlorophyll directly in response to drought stress^59^. Besides, chlorophyll b was more severely affected under drought stress than the other pigments, the reason is that chlorophyll b is more sensitive to osmotic stress as compared to chlorophyll b.^60^ Drought stress caused a severe reduction in biochemical accumulation such as soluble sugars, protein and phenolics contents and antioxidant machinery (Figure 2a-–f). Reduction in photosynthetic assimilates may be the main reason for lower production of these biomolecules,^57^ because lower ATP is available for the synthesis of these biochemicals. Besharati et al.^61^ also identified a lower accumulation of total soluble phenolics under severe drought stress, due to diminished photosynthetic activities. Lowest proline and protein content was found when thiamine was not applied, a similar trend was confirmed by Mehrasa et al.,^62^ while working on white bean under drought stress. However, thiamine application, especially at the dose of 500 ppm, significantly improved the secondary metabolites and antioxidant production, especially in sarsabz as compared with metior varieties as confirmed by Hosseinifard et al.^63^ Thiamine acts as signaling molecule under biotic and abiotic stress, and modulates the plant metabolism according to its environment.^64^ Vitamins could be considered as a compound of bioregulators or phytohormone precursors that, in a tiny amount, retain a beneficial effect on plant growth, development that could effect on the energy metabolic pathways and antioxidant production^65–67^ as confirmed in this study. So, all essential physiological process, secondary metabolites and nutrients are more and less dependent on the availability of vitamins.^68^ Similarly, in this study, higher dependence of nutrient uptake was seen on thiamine availability. These results are corroborated with the findings of Arafa et al.^19^, while working on pea as a model plant. However, lower nutrient accumulation was recorded under drought stress as shown in Figure 3a-–c. The lower uptake of the nutrients are due to the osmotic effect of drought stress.^54^ The PCA and pearson correlation matrix showed strong relation with sodium and proline (Figures 4 and 5), that might be due to the metabolic reshuffle during stress and osmotic effect of drought stress. Heatmap matrix showed positive correlation of sarsabz variety with almost all the above mentioned parameters as compared with metior variety.

## 5. Conclusion

**Figure 7.**
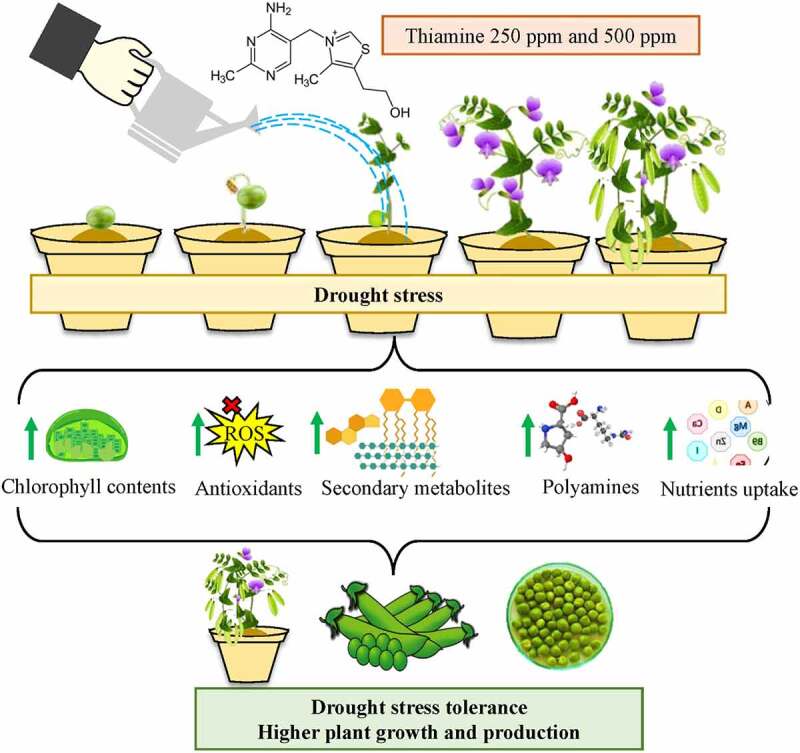
Mechanism of thiamine-induced drought stress tolerance in pea plants. In short, thiamine application improves drought tolerance by increasing several biochemical attributes such as chlorophyll contents, secondary metabolites, polyamines, and nutrient contents. Whereas it also helps ROS scavenging by increasing antioxidant enzyme activities. Upwards green means increased/upregulated, while downward green arrow mean decreased/downregulated.

Drought stress is an predictable factor that happens in multiple environments deprived of recognizing borders and no clear warnings, thereby hindering plant biomass and quality of agriculture production. However, foliar application of thiamine, especially 500 ppm, significantly improved photosynthetic attributes, soluble sugars, proline, pehnolics, antioxidants, and mineral uptake that resulted in higher growth and productivity irrespective of varietal differences (Figure 7); however, the performance of sarsabz variety was much better than metior variety. The beans are rich source of proteins and are mostly preferred due to their high nutritional profile. Thiamine application further enhances protein accretion up to 52–62.83%. The vitamin thiamine application offers a rich source for understanding many aspects of plants primary and secondary metabolism. Exogenous application of thiamine opens new avenues to boost the abiotic resistance mechanisms, especially against drought stress that could overcome the economic burden of poor productivity and treat and rehabilitate severely malnourished problems under climate variability.

## Authors contribution

The authors confirm their contribution to the paper as follows: study conception and design: A.K.; data collection: H.Z. and N.Z.; analysis and interpretation of results: M.B.H., A.R., N.Z., A.R., and S.Z.; draft manuscript preparation, proofreading and editing: A.K., A.F, N.Z, A.R, P.V.V.P., and I.D. All authors reviewed the results and approved the final version of the manuscript.

## Data Availability

The data reported in this study will be available from the corresponding author on a reasonable request.
